# *In Vitro* Activity of the Ultrabroad-Spectrum Beta-Lactamase Inhibitor QPX7728 in Combination with Multiple Beta-Lactam Antibiotics against Pseudomonas aeruginosa

**DOI:** 10.1128/AAC.00210-21

**Published:** 2021-05-18

**Authors:** Olga Lomovskaya, Debora Rubio-Aparicio, Kirk Nelson, Dongxu Sun, Ruslan Tsivkovski, Mariana Castanheira, Jill Lindley, Jeffery Loutit, Michael Dudley

**Affiliations:** a Qpex Biopharma, Inc., San Diego, California, USA; b JMI Laboratories, North Liberty, Iowa, USA

**Keywords:** QPX7728, beta-lactams, OprD, efflux, *Pseudomonas aeruginosa*, efflux pumps

## Abstract

QPX7728 is an ultrabroad-spectrum beta-lactamase inhibitor with potent inhibition of key serine and metallo beta-lactamases. QPX7728 enhances the potency of multiple beta-lactams in beta-lactamase-producing *Enterobacterales* and Acinetobacter spp. In this study, we evaluated the *in vitro* activity of QPX7728 (QPX; 8 μg/ml) combined with multiple beta-lactams against clinical isolates of Pseudomonas aeruginosa with various beta-lactam resistance mechanisms. Seven hundred ninety clinical isolates were included in this study; 500 isolates, termed a “representative panel,” were selected to be representative of the MIC distribution of meropenem (MEM), ceftazidime-avibactam (CAZ-AVI), and ceftolozane-tazobactam (TOL-TAZ) resistance for clinical isolates according to 2017 SENTRY surveillance data. An additional 290 selected isolates (“challenge panel”) that were either nonsusceptible to MEM or were resistant to TOL-TAZ or CAZ-AVI were also tested; 61 strains carried metallo-beta-lactamases (MBLs), 211 strains were defective in the carbapenem porin OprD, and 185 strains had the MexAB-OprM efflux pump overproduced based on a phenotypic test. Against the representative panel, susceptibility for all QPX7728/beta-lactam combinations was >90%. For the challenge panel, QPX-ceftolozane (TOL) was the most active combination (78.6% susceptible) followed by equipotent QPX-piperacillin (PIP) and QPX-cefepime (FEP), restoring susceptibility in 70.3% of strains (CLSI breakpoints for the beta-lactam compound alone). For MBL-negative strains, QPX-TOL and QPX-FEP restored the MIC values to susceptibility rates in ∼90% and ∼80% of strains, respectively, versus 68% to 70% for QPX-MEM and QPX-PIP and 63% to 65% for TOL-TAZ and CAZ-AVI, respectively. For MBL-positive strains, QPX-PIP restored the MIC to susceptibility values for ∼70% of strains versus 2% to 40% for other combinations. Increased efflux and impaired OprD had various effect on QPX7728 combination depending on the partner beta-lactam tested. QPX7728 enhanced the potency of multiple beta-lactams against P. aeruginosa, with varied results according to beta-lactamase production and other intrinsic resistance mechanisms.

## INTRODUCTION

Pseudomonas aeruginosa is a causative agent of various infections that are often life threatening. Acute infections such as pneumonia and sepsis, particularly in patients in intensive care units, are associated with high morbidity and lethality (1), and chronic airway infections with P. aeruginosa are a main comorbidity in patients with cystic fibrosis (2), bronchiectasis (3). and chronic obstructive pulmonary disease (COPD) (4).

Due to efficient efflux activity combined with the low permeability of the outer membrane, P. aeruginosa is intrinsically resistant to a large number of marketed antibiotics (5–8). Acquired resistance to this already limited arsenal of available antibiotics further threatens the successful management of *Pseudomonas* infections (9). Acquired resistance is often a result of chromosomal mutations resulting in overexpression of efflux pumps (affecting multiple antibiotics) or chromosomal class C beta-lactamase (multiple beta-lactams), inactivation of porin OprD (carbapenems), target mutations (fluoroquinolones), or outer membrane modification (polymyxins) (10). It also can be due to horizontal uptake of mobile genomic islands and integrons encoding carbapenemases or extended-spectrum β-lactamases, both of particular concern (11). The Centers for Disease Control and Prevention lists multidrug-resistant *Pseudomonas* as a serious threat (12), and the World Health Organization recognizes carbapenem-resistant Pseudomonas aeruginosa as a pathogen for which new agents are critically needed (13).

Three novel beta-lactam (BL)–beta-lactamase inhibitor (BLI) combination products, ceftazidime-avibactam, ceftolozane-tazobactam, and imipenem-relebactam, and first-in-class siderophore cephalosporin, cefiderocol, were recently approved for clinical use with activity against P. aeruginosa (14–16). Avibactam and relebactam are inhibitors of serine beta-lactamases, with neither of them having inhibitory activity against metalloenzymes (14). While cefiderocol has potent *in vitro* activity against nonfermenting bacteria, two clinical studies showed poor clinical efficacy in systemic infections due to Acinetobacter and, potentially, Pseudomonas aeruginosa (15, 16). Hence, ongoing research and development aiming to identify improved approaches for treatment of these pathogens is warranted.

QPX7728 is a recently discovered ultrabroad-spectrum beta-lactamase inhibitor from a class of cyclic boronates (17). It has two major improvements in spectrum compared to the currently marketed agents (diazabicyclooctane [DBO] avibactam and relebactam and cyclic boronate vaborbactam) by being an efficient inhibitor of class D carbapenemases from Acinetobacter baumannii such as OXA-23, OXA-24/40, and OXA-58 and by inhibiting various class B metallo-beta-lactamases from the B1 subclass such as NDM, VIM, and IMP (18). QPX7728 restores the potency of multiple beta-lactam antibiotics in strains producing these beta-lactamases (19, 20). Combinations of QPX7728 with cephalosporins or carbapenems, including meropenem, were found to have excellent *in vitro* activity against carbapenem-resistant *Enterobacterales* producing either serine or metallo-beta-lactamases with or without permeability defects (21). QPX7728 combinations with meropenem or sulbactam were highly potent against carbapenem-resistant Acinetobacter baumannii (22, 23). QPX7728 also enhances the activity of multiple beta-lactams *in vivo* in a mouse thigh infection model due to Klebsiella pneumoniae carbapenemase (KPC)-producing Klebsiella pneumoniae (24). In this study, we investigated the potency of QPX7728 in combination with multiple beta-lactam antibiotics against P. aeruginosa, including strains resistant to recently approved BL/BLI combinations.

## RESULTS AND DISCUSSION

Using large panels of clinical isolates of carbapenem-resistant *Enterobacterales* and Acinetobacter baumannii, we previously identified the QPX7728 concentration required to shift meropenem MICs for >90% of isolates to ≤8 μg/ml; this value corresponds to the meropenem pharmacokinetic/pharmacodynamic (PK/PD) breakpoint for 2 g meropenem administered as a 3-h intravenous (i.v.) infusion every 8 h. This concentration of QPX7728, called 90% target potentiation concentration (TPC_90_) was found to be ≤8 μg/ml (21, 23). The plasma QPX7728 exposure associated with these concentrations was associated with efficacy in animal models of infection (24), safe in preclinical studies, and will be targeted in human dosage regimens. Thus, for purposes of the analyses below, a fixed concentration of 8 μg/ml of QPX7728 was chosen for *in vitro* susceptibility testing in combination with multiple antibiotics against Pseudomonas aeruginosa.

### QPX7728 with meropenem, cefepime, or ceftolozane results in highly potent combinations against clinical isolates of Pseudomonas aeruginosa representative of the current MIC distributions for these beta-lactams.

The *in vitro* activity of meropenem, ceftolozane, or cefepime combined with QPX7728 at 8 μg/ml was determined against the panel of 500 isolates of Pseudomonas aeruginosa that were selected to represent the MIC distributions of meropenem and ceftazidime-avibactam from the 2017 SENTRY surveillance data (“representative panel”) (see Fig. S1 in the supplemental material). Greater than 90% of these isolates were also susceptible to ceftolozane-tazobactam and ceftazidime-avibactam (25).

All QPX7728 combinations had excellent potency against the representative panel. QPX7728 decreased the meropenem MIC_90_ 2-fold, from 16 μg/ml to 8 μg/ml. The MIC_90_ of cefepime was decreased 4-fold by QPX7728, from 32 μg/ml to 8 μg/ml. Ceftolozane alone (or with tazobactam) MIC_90_ values were decreased 4-fold (i.e., 4 μg/ml to 1 μg/ml). Greater than 90% of isolates were inhibited at 8 μg/ml of meropenem and cefepime or at 4 μg/ml of ceftolozane when combined with QPX7728 (Table 1).

**TABLE 1:** *In vitro* potency of meropenem, ceftolozane, and cefepime alone and combined with QPX7728 at 8 μg/ml and comparator agents against representative panel of clinical isolates of Pseudomonas aeruginosa (*n* = 500)

Drugs^*a*^	MIC (μg/ml)		% inhibited^*b*^
	50%	90%	
MEM	0.5	16	84.8
QPX+MEM	0.25	8	91.6
TOL	0.5	4	90.4
TOL+TAZ	0.5	4	91.8
QPX+TOL	0.5	1	97.6
FEP	4	32	74.4
QPX+FEP	2	8	91.2
CAZ+AVI	2	8	92.2
PIP+TAZ	8	128	71.6

a MEM, meropenem; QPX, QPX7728; TOL, ceftolozane; TAZ, tazobactam; FEP, cefepime; CAZ, ceftazidime; AVI, avibactam; PIP, piperacillin; TAZ and AVI were tested at a fixed 4 μg/ml. QPX was tested at a fixed at 8 μg/ml.

b Percent inhibited at the following concentrations: MEM, ≤8 μg/ml; QPX+MEM, ≤8 μg/ml with QPX at 8 μg/ml; TOL, ≤4 μg/ml; QPX+TOL, ≤4 μg/ml with QPX at 8 μg/ml; TOL+TAZ, ≤4/4 μg/ml (FDA susceptible breakpoint); FEP, ≤8 μg/ml (FDA susceptible breakpoint); QPX+FEP, ≤8 μg/ml with QPX at 8 μg/ml; CAZ+AVI, ≤8/4 μg/ml (FDA susceptible breakpoint); PIP+TAZ, ≤16 μg/ml (FDA susceptible breakpoint).

### QPX7728 in combination with several beta-lactams is more potent than recently approved BLI combinations against the challenge panel of resistant P. aeruginosa.

*In vitro* potency of meropenem, ceftolozane, cefepime, piperacillin, and aztreonam in combination with QPX7728 (8 μg/ml) was also determined against a panel of 290 isolates that were either nonsusceptible to meropenem (MIC ≥ 4 μg/ml) or ceftolozane-tazobactam (MIC ≥ 8/4 μg/ml) or resistant to ceftazidime-avibactam (MIC ≥ 16/4 μg/ml).

Susceptibility to BL/BLI combinations approved for P. aeruginosa were 51% to 52% for ceftolozane-tazobactam and ceftazidime-avibactam, ∼47% for imipenem-relebactam, and 17% for piperacillin-tazobactam. For meropenem, at a susceptibility breakpoint of 2 μg/ml, the susceptibility was only ∼3% but increased to 33% of isolates using a susceptibility breakpoint of 8 μg/ml (based on 2 g of meropenem administered every 8 h as a 3-h infusion [26–28]) (Table 2).

**TABLE 2:** *In vitro* potency of various beta-lactams combined with QPX7728 at 8 μg/ml and comparator BLI combination agents against the challenge panel of clinical isolates of Pseudomonas aeruginosa

Drug(s)^*a*^	All (*n* = 290)			No MBL (*n* = 229)			MBL (*n* = 61)		
	MIC (μg/ml)		% inhibited^*b*^	MIC (μg/ml)		% inhibited^*b*^	MIC (μg/ml)		% inhibited^*b*^
	50%	90%		50%	90%		50%	90%	
MEM	16	>64	33.1	16	64	41.9	>64	>64	0.0
QPX+MEM	8	64	60.3	8	32	68.1	32	>64	31.1
ATM	32	>64	19.0	32	>64	17.9	32	>64	23.0
QPX+ATM	16	64	45.9	16	32	46.7	16	64	42.6
PIP	>64	>64	16.2	>64	>64	19.7	>64	>64	3.3
QPX+PIP	16	64	70.3	16	64	69.9	16	64	72.1
TOL	4	>64	51.4	2	>64	64.2	>64	>64	3.3
QPX+TOL	1	64	78.6	1	4	92.1	64	>64	27.9
FEP	32	64	20.0	32	64	23.6	64	64	6.6
QPX+FEP	8	64	70.3	8	16	79.5	32	64	36.1
PIP+TAZ	>64	>64	17.2	>64	>64	20.1	>64	>64	6.6
TOL+TAZ	4	>64	52.1	2	>64	65.5	>64	>64	1.6
CAZ+AVI	8	>64	50.7	8	64	62.9	>64	>64	4.9
ATM+AVI	16	64	33.8	16	64	33.6	16	64	34.4
IMP+REL	4	64	46.9	2	16	59.0	>64	>64	1.6

a MEM, meropenem; QPX, QPX7728; TOL, ceftolozane; ATM, aztreonam; TAZ, tazobactam; FEP, cefepime; CAZ, ceftazidime; AVI, avibactam; PIP, piperacillin; IMP, imipenem; REL, relebactam. TAZ and AVI were tested at a fixed 4 μg/ml. QPX was tested at a fixed 8 μg/ml.

b Percent inhibited at the following concentrations: MEM, ≤8 μg/ml; QPX+MEM, ≤8/8 μg/ml; ATM, ≤8 μg/ml (FDA susceptible breakpoint); QPX+ATM, ≤8/8 μg/ml; TOL, ≤4 μg/ml; QPX+TOL, ≤4/8 μg/ml; TOL+TAZ, ≤4/4 μg/ml (FDA susceptible breakpoint); FEP, ≤8 μg/ml (FDA susceptible breakpoint); QPX+FEP, ≤8/8 μg/ml; CAZ+AVI, ≤8/4 μg/ml (FDA susceptible breakpoint); PIP+TAZ, ≤16/4 μg/ml (FDA susceptible breakpoint); IMP+REL, ≤2/4 μg/ml (FDA susceptible breakpoint).

**FIG 1:**
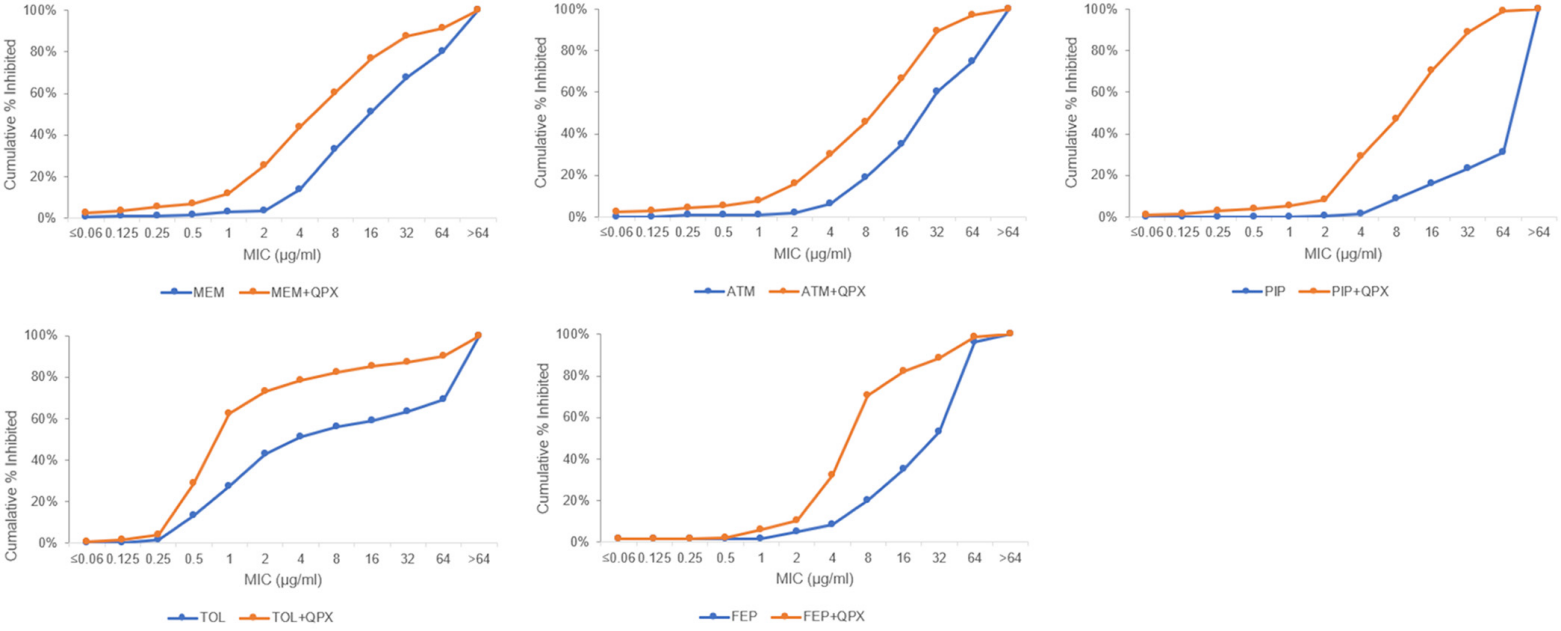
The effect of QPX7728 (8 μg/ml) on the *in vitro* potency of several antibiotics against the challenge panel of Pseudomonas aeruginosa isolates (*n* = 290). MEM, meropenem; ATM, aztreonam; PIP, piperacillin; TOL, ceftolozane; FEP, cefepime; QPX, QPX7728.

QPX7728 increased the *in vitro* potency of all beta-lactams against the challenge panel (Fig. 1). All tested QPX7728 combinations, except for QPX7728 (QPX)-aztreonam (ATM), were more active than marketed combinations based on the percentage of strains inhibited at the CLSI breakpoint for the compound alone. QPX-ceftolozane (TOL) was the most active combination overall, with 4 μg/ml of TOL inhibiting 78.6% of isolates. This combination was followed by QPX-cefepime (FEP), QPX-piperacillin (PIP) (70.3% inhibited at MIC of ≤8 μg/ml and ≤16 μg/ml, respectively), and QPX-meropenem (MEM) (65.5% inhibited at MIC of ≤8 μg/ml) (Table 2).

### The activity of QPX7728 combinations depends on the presence or absence of MBLs.

**FIG 2:**
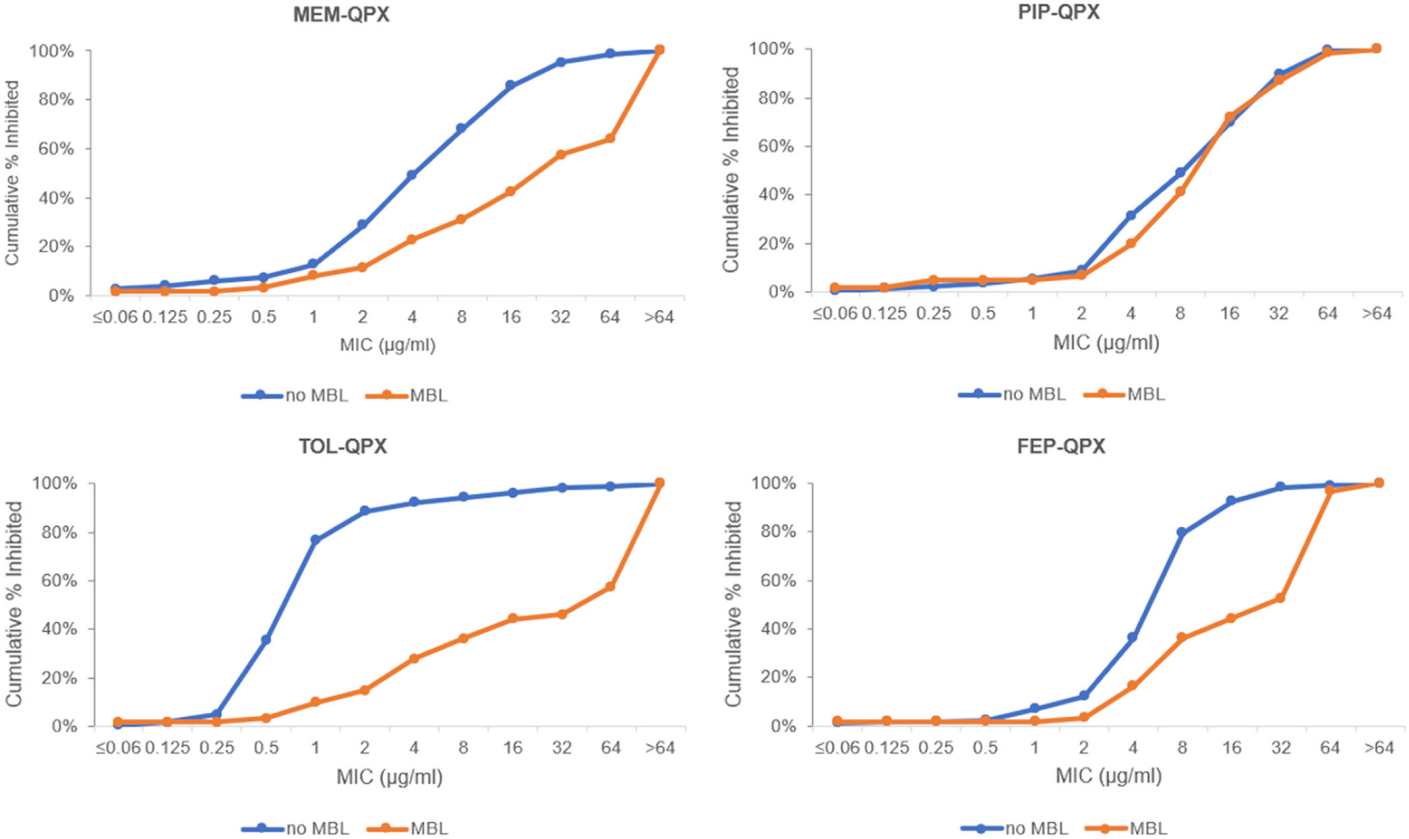
*In vitro* potency of various beta-lactam antibiotics in combination with QPX7728 (8 μg/ml) against MBL-negative (*n* = 229) and MBL-positive (*n* = 61) clinical isolates of Pseudomonas aeruginosa. MEM, meropenem; ATM, aztreonam; PIP, piperacillin; TOL, ceftolozane; FEP, cefepime; QPX, QPX7728.

QPX7728 increased potency of all tested antibiotics against metallo-beta-lactamase (MBL)-negative and MBL-positive isolates (Table 2). QPX-MEM, QPX-FEP, and QPX-TOL were more potent against MBL-negative strains, while the potency of QPX-ATM and QPX-PIP was similar in MBL-and non-MBL-producing isolates (Fig. 2). Against the subset of 229 MBL-negative strains in the challenge panel, the most potent combination was QPX-TOL; the MIC_90_ of ceftolozane was reduced >16-fold by QPX7728, (>64 μg/ml to 4 μg/ml), with the susceptibility of ceftolozane (at ≤4 μg/ml) increasing from 64% to 92%. The second most potent combination was QPX-FEP; the MIC_50_/MIC_90_ values of cefepime were reduced 4-fold and susceptibility (at ≤8 μg/ml) was increased from 23.6% to 79.5%. QPX-PIP and QPX-MEM susceptibility increased from 19.7% to 69.9% and from 41.9% to 68.1%, respectively, at breakpoints of ≤16 μg/ml and ≤8 μg/ml for piperacillin and meropenem, respectively. Against this subset of strains, the least potent QPX7728 combination was QPX-ATM, with susceptibility of only 46.7% (at 8 μg/ml). For the MBL-negative subset, susceptibility of ceftolozane-tazobactam, ceftazidime-avibactam, and imipenem-relebactam ranged from 59% to 65.5% (Table 2).

Notably, when tested against 61 MBL-positive strains in the challenge panel, QPX-TOL was the least active QPX7728 combination (27.9% of coverage at breakpoint of 4 μg/ml), and QPX-PIP covered the highest percentage of strains at the piperacillin breakpoint (72.1%). The susceptibility of other QPX7728 combinations ranged from 27.9% to 42.6%, and was markedly higher than that for ceftazidime-avibactam, ceftolozane-tazobactam, or imipenem-relebactam (1.6% to 4.9%) (Table 2).

To better understand the PIP/TOL discordance observed for MBL producers, we looked at the impact of different breakpoints. Our data indicate that if TOL had a breakpoint of 16 μg/ml like PIP, the coverage would be 44.3% (Fig. 2). Hence, while the difference will be ∼10% less, different breakpoints will not explain all the difference.

Previous results with QPX7728 combinations against the strains producing cloned metallo-beta-lactamases (20) showed that MIC values with piperacillin alone and QPX-PIP were the lowest and those with ceftolozane alone and QPX-TOL were the highest. We hypothesize that potentiation of PIP by QPX7728 appears to be more efficient than that of TOL (and other beta-lactams) because PIP is less labile to hydrolysis by MBLs than TOL (or other beta-lactams). According to this hypothesis, the activity of QPX7728 combinations against MBL-positive clinical isolates can be explained in part by the differential susceptibility of the partner beta-lactam to MBL-mediated hydrolysis. High PIP MIC (>64 μg/ml) observed for the majority of MBL producers is due to coproduction of various serine beta-lactamases (overexpressed chromosomal AmpC and various extended-spectrum beta-lactamases [ESBLs]) that are inhibited by QPX7728 (data not shown).

### The activity of different QPX7728/beta-lactam combinations varies according to intrinsic non-beta-lactamase-mediated resistance mechanisms of P. aeruginosa that primarily affect the partner antibiotic.

We hypothesize that in addition to the type of beta-lactamase production and the inhibitory profile of QPX7728, non-beta-lactamase-mediated resistance mechanisms modulate the potency of various QPX7728-beta-lactam combinations. A panel of isogenic strains of P. aeruginosa with various combinations of efflux and uptake mutations was used to assess the effects of these intrinsic mechanisms on the potency of beta-lactams used in this study (Table 3). Our results were in good agreement with published data (5, 7, 29–31) and indicate that (i) ceftolozane is not effluxed by any of the major multidrug resistance efflux pumps; (ii) meropenem, aztreonam, and piperacillin are effluxed by MexAB-OprM and cefepime is effluxed by the MexAB-OprM, MexXY-OprM, and MexCD-OprJ efflux pumps and that, notably, MIC values for aztreonam and piperacillin are raised to or above their susceptibility breakpoints; (iii) MICs for the carbapenem meropenem, but not for ceftolozane, cefepime, aztreonam, or piperacillin, are increased against the strains defective in the porin OprD; and (iv) MexAB-OprM efflux and OprD inactivation have a multiplicative effect on the meropenem MIC, raising it to or above the PK/PD breakpoint of 8 μg/ml (Table 3). Hence, ceftolozane was affected the least by these mechanisms, followed by cefepime, meropenem, piperacillin, and aztreonam. This rank order basically mirrors the ranking of QPX7728 combinations with these antibiotics; this indeed makes it quite plausible that the potency of various QPX7728 combinations and its ranking observed against MBL-negative strains might be a consequence of the differential impact of various intrinsic non-beta-lactamase-mediated resistance mechanisms of P. aeruginosa on the potency of different beta-lactams alone. This conclusion is in a good agreement with our earlier study that demonstrated that the whole-cell beta-lactamase-potentiating activity of QPX7728 was not affected by either increased efflux or defects in OprD (19).

**TABLE 3:** *In vitro* potency of various beta-lactams against the isogenic strains of Pseudomonas aeruginosa containing various combinations of intrinsic resistance mechanisms relevant to beta-lactam antibiotics

Strain^*a*^	Genotype	Description^*b*^	MIC (μg/ml)							
			Meropenem	Aztreonam	Ceftolozane	Ceftazidime	Cefepime	Piperacillin	Levofloxacin	Amikacin
PAM1020	Wild type	Wild type	0.5	4	0.5	1	0.5	4	0.25	2
PAM1101	*oprD1101*	OprD inactivated	4	4	0.5	1	0.5	4	0.25	2
PAM1032	*mexR*::L75R	MexAB-OprM up	2	32	1	4	4	16	2	2
PAM1106	*mexA*::Tet	MexAB-OprM inactivated	0.125	0.125	0.5	0.5	0.25	0.125	0.06	2
PAM1154	*oprM*::Hg	MexAB-OprM and MexXY-OprM inactivated	0.125	0.125	0.5	0.5	0.125	0.125	0.015	0.5
PAM2156	*ampR*::D135H	AmpC up	2	64	4	32	8	>128	0.25	2
PAM2005	*mexR*::L75R *ampR*::D135H	MexAB up, AmpC up	4	64	4	32	8	>128	2	2
PAM2059	*mexR*::L75R *oprD2059*	MexAB up, OprD inactivated	8	32	1	4	4	16	2	2
PAM2091	*mexR*::L75R *ampR*::D135H *oprD2091*	MexAB up, AmpC up, OprD inactivated	16	64	4	32	16	>128	2	2
PAM1033	*nfxB*::T39I	MexCD-OprJ up	0.25	1	0.5	0.5	4	2	4	0.25
PAM1034	mexT::P242R	MexEF-OprN up	1	2	0.5	1	0.5	4	8	1
PAM1323	mexZ::Δ(aa25 to 39)	MexXY-OprM up	0.5	4	0.5	1	2	4	0.5	4

a All strains are derivatives of PAM1020 (PAO1). PAM1101 was selected from PAM1020 on 4 μg/ml of imipenem and carries a 198-bp deletion from −36 bp to +162 bp of *oprD* ATG; PAM2059 was selected from PAM1032 on 4 μg/ml of imipenem and carries TAA at amino acid (amino acid) number 399 of OprD; PAM2091 was selected from PAM2005 on 4 μg/ml of imipenem and has a frameshift from aa 407 of OprD. Selection or construction of efflux mutants is described in reference 37.

b up, overexpressed.

To better understand how the interplay of various resistant mechanisms affects the potency of specific QPX7728-beta-lactam combinations, we looked at their potency against several subsets of strains stratified according to resistance mechanisms.

### In MBL-negative strains from the challenge panel, susceptibility of the partner beta-lactam to the MexAB-mediated efflux is an important factor that determines the activity of QPX7728 combinations.

All QPX7728 combinations were highly active (>93% inhibited) against the 77 isolates with low activity of MexAB-OprM. Increased activity of the MexAB-OprM pump was detected in 145 strains; QPX-TOL and QPX-FEP were the least affected and, hence, the most active combinations inhibiting 89.7% and 72.4% of isolates, respectively, against this subset of strains. QPX-MEM and QPX-PIP inhibited 52% to 54% of these strains, and QPX-ATM inhibited just 18% (Table 4).

**TABLE 4:** *In vitro* potency of various beta-lactams combined with QPX7728 at 8 μg/ml against the isolates from the challenge panel of Pseudomonas aeruginosa according to presence or absence of MBL and efflux due to MexAB-OprM

Drug(s)^*a*^	No MBL (*n* = 229)			No MBL, MexAB-OprM basal-level activity (*n* = 77)			No MBL, MexAB-OprM increased activity (*n* = 45)			MBL (*n* = 61)			MBL, MexAB-OprM basal-level activity (*n* = 21)			MBL, MexAB-OprM increased activity (*n* = 40)		
	MIC (μg/ml)		% inhibited^*b*^	MIC (μg/ml)		% inhibited^*b*^	MIC (μg/ml)		% inhibited^*b*^	MIC (μg/ml)		% inhibited^*b*^	MIC (μg/ml)		% inhibited^*b*^	MIC (μg/ml)		% inhibited^*b*^
	50%	90%		50%	90%		50%	90%		50%	90%		50%	90%		50%	90%	
MEM	16	64	41.9	8	64	70.1	16	64	25.5	>64	>64	0.0	64	>64	0.0	>64	>64	0.0
QPX+MEM	8	32	68.1	2	4	96.1	8	32	52.4	32	>64	31.1	4	64	66.7	64	>64	12.5
ATM	32	>64	17.9	16	>64	44.2	64	>64	0.0	32	>64	23.0	8	32	66.7	32	>64	0.0
QPX+ATM	16	32	46.7	4	8	96.1	32	64	17.9	16	64	42.6	2	8	95.2	32	64	15.0
PIP	>64	>64	19.7	>64	>64	35.1	>64	>64	10.3	>64	>64	3.3	>64	>64	9.5	>64	>64	0.0
QPX+PIP	16	64	69.9	4	8	97.4	16	64	54.5	16	64	72.1	4	16	90.5	16	64	62.5
TOL	2	>64	64.2	2	>64	67.5	4	>64	60.7	>64	>64	3.3	>64	>64	4.8	>64	>64	2.5
QPX+TOL	1	4	92.1	0.5	1	96.1	1	8	89.7	64	>64	27.9	4	>64	52.4	>64	>64	15.0
FEP	32	64	23.6	16	64	39.0	32	64	15.2	64	64	6.6	64	64	9.5	64	64	5.0
QPX+FEP	8	16	79.5	4	8	93.5	8	32	72.4	32	64	36.1	8	64	52.4	64	64	27.5

a MEM, meropenem; QPX, QPX7728; ATM, aztreonam; TOL, ceftolozane; FEP, cefepime; PIP, piperacillin.

b Percent inhibited at the following concentrations: MEM, ≤8 μg/ml; QPX+MEM, ≤8/8 μg/ml; ATM, ≤8 μg/ml (FDA susceptible breakpoint); QPX+ATM, ≤8/8 μg/ml; TOL, ≤4 μg/ml; QPX+TOL, ≤4/8 μg/ml; FEP, ≤8 μg/ml (FDA susceptible breakpoint); QPX+FEP, ≤8/8 μg/ml. QPX was tested at a fixed 8 μg/ml.

Of 145 isolates with increased efflux, 105 had a nonfunctional OprD. In good agreement with previous data from the isogenic strain panel, only the potency of QPX-MEM but no other QPX7728 combinations was impacted by inactivation of OprD (see Table S2). For QPX-MEM, inactivation of OprD in the strains with increased MexAB-OprM-mediated efflux resulted in a decrease in the proportion of strains inhibited at ≤8 μg/ml of meropenem from 52.4% to 40%. However, QPX-MEM inhibited 81.8% and 94.6% of isolates that had increased activity of MexAB-OprM and functional OprD or constitutive activity of MexAB-OprM and nonfunctional OprD, respectively (Table 5).

**TABLE 5:** *In vitro* potency of QPX7728-meropenem against MBL-negative and MBL-positive isolates from the challenge panel according to presence of functioning or nonfunctioning OprD and efflux due to MexAB-OprM alone or in combination

Condition^*a*^	MBL negative				MBL positive			
	No.	MIC (μg/ml)		% inhibited	No.	MIC (μg/ml)		% inhibited
		50%	90%			50%	90%	
Total	229	8	32	68.1	61	32	>64	31.1
MexAB wt	77	2	4	96.1	21	4	64	66.7
MexAB up	145	8	32	52.4	40	64	>64	12.5
OprD F	47	4	16	87.2	11	8	64	63.6
OprD NF	163	8	32	58.9	48	32	>64	25.0
MexAB wt, OprD F	12	1	4	100.0	6	1	ND^*b*^	ND
MexAB up, OprD F	33	8	16	81.8	5	32	ND	ND
MexAB wt, OprD NF	56	2	8	94.6	15	8	>64	60.0
MexAB up, OprD NF	105	16	32	40.0	33	>64	>64	9.1

a wt, wild type (constitutive); up, overexpressed; F, functional; NF, nonfunctional.

b ND, not done.

In summary, (i) the coverage by QPX-MEM was driven by the proportion of strains that had both OprD inactivated and MexAB-OprM overproduced (∼50% strains in our challenge panel) (this combination still covered >80% of strains with either nonfunctional OprD or overproduced MexAB-OprM); (ii) the coverage by QPX-ATM and QPX-PIP was significantly affected by overproduction of MexAB-OprM alone; (iii) the effect of MexAB-OprM overproduction on the coverage by QPX-FEP was less significant and comparable to that of QPX-MEM (in the absence of *oprD* mutations); and (iv) neither uptake nor efflux appeared to affect the coverage by QPX-TOL.

### In MBL-positive strains in the challenge panel, the susceptibility of beta-lactams to MBL-mediated hydrolysis and efflux determines the activity of QPX7728 combinations.

QPX-PIP was the most potent combination against the MBL-positive strains overall (Table 4); however, the susceptibility of beta-lactams to MBL-mediated hydrolysis and efflux were two major factors affecting the potency of a specific QPX7728 combination. Accordingly, QPX7728 in combination with aztreonam, which is not hydrolyzed by MBLs, was the most active combination against the subset of MBL-positive strains with constitutive expression of MexAB-OprM. PIP, which is hydrolyzed by MBLs relatively poorly, had 90% of isolates inhibited in combination with QPX7728. Coverage by other combinations ranged from 52% for QPX-FEP and QPX-TOL to 66% for QPX-MEM. Against MBL-positive MexAB-overproducing strains, QPX-PIP was the most active combination, but the coverage decreased from 95% to ∼60% due to the impact of MexAB. All other combinations, including QPX-ATM, had coverage ranging from 12.5% to 27.5%.

### Differential susceptibility to different QPX7728 combinations can be detected at the individual strain level.

Examination of the susceptibility of individual strains to various QPX7728 combinations demonstrated that several strains exist that are susceptible to a single unique QPX7728 combination. These strains are summarized in Table 6. Four strains were susceptible to QPX-TOL only (two of them were also intermediate with QPX-FEP), four strains were susceptible to QPX-FEP only (all of them intermediate for other combinations), six strains were susceptible only to QPX-PIP, and 2 strains were susceptible only to QPX-ATM. Thus, the optimal beta-lactam for use in combination with QPX7728 can be identified by testing multiple beta-lactams as well as understanding the interplay of various intrinsic resistance mechanisms.

**TABLE 6:** *In vitro* potency of various beta-lactams combined with QPX7728 at 8 μg/ml against the individual isolates from the challenge panel of Pseudomonas aeruginosa^*a*^

Strain	OprD status	MexAB-OprM status	Acquired beta-lactamase	MIC (μg/ml)				
				QPX-MEM	QPX-ATM	QPX-PIP	QPX-TOL	QPX-FEP
PA5314	FL	Up	IMP-14	32	32	**16**	>64	>64
PA5271	NF	Up	IMP-7	>64	32	**16**	>64	64
PA5274	NF	Up	IMP-1	>64	32	**16**	>64	64
PA5474	NF	Up	IMP-48	>64	32	**16**	64	>64
PA5487	NF	Up	IMP-14, OXA-10	>64	32	**16**	>64	>64
PA5311	NF	Up	IMP-6	>64	64	**16**	>64	>64
PAM3267	NF	Up	VIM-5	32	16	**32**	64	**8**
PA5436	NF	Up	VEB-9	*16*	32	**32**	32	**8**
PA5388	NF	Up	VIM-2;	32	64	**32**	16	**8**
PA5282	NF	Up	CTX-M-3	*16*	32	64	32	**8**
PA5376	FL	Wt	NDM-1	64	**4**	64	>64	>64
PA1068	NF	Wt	SPM-1	>64	**4**	64	>64	>64
PA5426	NF	Up	ND	32	32	64	**2**	32
PA5309	NF	Up	ND	32	64	64	**1**	*16*
PA5369	NF	Up	ND	64	64	64	**2**	*16*
PA5459	NF	Up	ND	64	>64	64	**1**	32

a QPX, QPX7728 tested at a fixed concentration of 8 μg/ml. Numbers in boldface font correspond to MICs in the susceptible range based on the FDA-approved breakpoints for antibiotics alone. Numbers in italics correspond to MICs in the intermediate range. For QPX-MEM, susceptible MIC and intermediate MIC were assumed to be 8 and 16 μg/ml, respectively (based on 2 g every 8 h as a 3-h infusion).

### Summary and conclusions.

QPX7728 enhanced the potency of multiple beta-lactams against a representative panel of P. aeruginosa isolates that reflects the current beta-lactam MIC distributions. These data indicate that QPX7728 would be expected to be useful in combination with multiple partner beta-lactams and would enhance their susceptibility at current breakpoints. This supports the notion that multiple beta-lactams would benefit from addition of a broad-spectrum beta-lactamase inhibitor and help to preserve the usefulness of beta-lactam antibiotics in multiple settings.

QPX7728 also enhanced the potency of multiple beta-lactams against a challenge panel of P. aeruginosa enriched with ceftazidime-avibactam-resistant and ceftolozane-tazobactam- or meropenem-nonsusceptible isolates. Several QPX7728 combinations were more potent than currently marketed combinations using tazobactam, avibactam, and relebactam. Overall, QPX-TOL was the most active combination against the challenge panel of isolates that did not produce metallo-beta-lactamases, while QPX-PIP was the most potent combination against MBL producers. QPX-FEP and QPX-MEM inhibited similar proportions of MBL or non-MBL-producing isolates from this challenge panel.

The activity of a specific QPX7728 combination depended on both the efficiency of beta-lactamase-mediated hydrolysis of a specific beta-lactam and its inhibition by QPX7728 and the impact of non-beta-lactamase-mediated beta-lactam resistance mechanisms. These studies have utility for decision making based on local epidemiology but can also be useful for individualization of treatment in a given patient with infection due to drug-resistant Pseudomonas aeruginosa.

These findings encourage serious considerations for a strategy that enables use of a broad-spectrum BLI in combination with multiple different beta-lactam antibiotics, particularly in patients infected with P. aeruginosa. In this approach, QPX7728 could be coadministered with different beta-lactam antibiotics, depending on the mechanisms present in the specific pathogen, providing clinicians and stewardship teams with greater optionality to treat highly resistant organisms with various resistance mechanisms. The flexibility of a stand-alone BLI, in conjunction with rapid identification and susceptibility testing, or patient factors, may be an integral component for implementation.

## MATERIALS AND METHODS

### Bacterial strains.

A worldwide collection of 500 nonduplicate clinical isolates of Pseudomonas aeruginosa was selected to represent the normal distribution of meropenem and ceftazidime-avibactam resistance according to the 2017 SENTRY Antimicrobial Surveillance Program (representative panel). An additional 290 isolates were selected from the Qpex collection of clinical isolates (originated from various surveillance studies) and were either nonsusceptible to meropenem or to ceftolozane-tazobactam or resistant to ceftazidime-avibactam (challenge panel). Two hundred of 290 isolated were collected in 2016 to 2018. Beta-lactamases were previously characterized in 200 strains of the 290 strains within the challenge panel either by PCR (80 strains) or by whole-genome sequencing (120 strains). Sixty-one strains carried metallo-beta-lactamases (MBLs; 31, VIM; 25, IMP; 3, NDM; 2, SPM), 23 strains carried class A carbapenemases (11, KPC; 13, GES-type, two of them in combination with MBLs), 43 strains carried varies ESBL enzymes (CTX-M, VEB, PER, GES, and SHV; seven of them in combination with MBLs), and 14 strains carried narrow-spectrum beta-lactamases (TEM-1, PSE-2, and OXA-10). The sequence of OprD was determined for 270 strains, and 211 of them were found to be defective in the carbapenem porin OprD. One hundred eighty-five of 290 strains had the MexAB-OprM efflux pump overproduced based on phenotypic test.

### Antimicrobial susceptibility testing.

Bacterial isolates were subjected to broth microdilution susceptibility testing, performed according to Clinical and Laboratory Standards Institute (CLSI) methods (32), using panels prepared in-house. MIC data for the representative panel were based on single tests; data for the challenge panel is the mode of a minimum of three experiments for the majority of BL/BLI combinations (and up to five, for some combinations). Meropenem was purchased from Carbosynth (San Diego, CA), and ceftolozane was synthesized at ACME Bioscience (Exton, PA); all other antibiotics and tazobactam were from Sigma-Aldrich. All beta-lactamase inhibitors were synthesized at Qpex Biopharma, San Diego, CA.

### Phenotypic testing for MexAB-OprM activity.

Antibiotic aztreonam is a substrate of the MexAB-OprM efflux pump, which is the major constitutively expressed multidrug resistance pump in P. aeruginosa. Multiple early studies, including those presented here, demonstrate (Table 3) that in the absence of overexpression of MexAB-OprM, aztreonam MICs do not exceed 8 μg/ml. At the same time, overexpression of MexAB-OprM (occurs due to various regulatory mutations [33–35]) results in aztreonam MICs ranging from 16 to 64 μg/ml. Since aztreonam is also susceptible to beta-lactamase hydrolysis and has an increased MIC against beta-lactamase-producing strains, its MIC alone cannot be used to assess MexAB-OprM overexpression. However, when beta-lactamases are inhibited with beta-lactamase inhibitors, the major factor that defines aztreonam MIC is the level of activity of MexAB-OprM. As aztreonam is stable to hydrolysis by metallo-beta-lactamases, aztreonam MIC measured in the presence of a potent inhibitor of class C and class A enzymes such as avibactam can be used to determine activity of MexAB-OprM even in the strains that produce MBLs. According to this phenotypic test, MIC values of aztreonam plus avibactam (4 μg/ml) of ≤8 μg/ml are predictive of MexAB-OprM activity associated with constitutive expression of the *mexAB-OprM* operon, while MIC values of aztreonam-avibactam (4 μg/ml) of ≥16 μg/ml are predictive of increased activity of MexAB-OprM due to overexpression of *mexAB-oprM*. The data validating this phenotypic test are presented in Table S1 in the supplemental material. The potential limitation of the test is that mutations in PBP3 that are associated with an increase in the ceftazidime-avibactam MIC (36) might also increase the aztreonam-avibactam MIC above 8 μg/ml, even in the absence of active efflux.
