# Supplementary material for: In Vitro Activity of the Ultrabroad-Spectrum Beta-Lactamase Inhibitor QPX7728 in Combination with Multiple Beta-Lactam Antibiotics against Pseudomonas aeruginosa
Source: Antimicrob Agents Chemother. 2021 May 18;65(6):e00210-21. doi: 10.1128/AAC.00210-21 (PMC8315991; doi:10.1128/AAC.00210-21)
Supplement: Supplementary file 1 [file aac.00210-21-s0001.pdf]

Supplementary Material

Figure S1. Distribution of meropenem and ceftazidime-avibactam MIC values using the panel of *P. aeruginosa* isolates collected by the 2017 SENTRY surveillance program (N=2910) and the representative panel used in this study (N=500)

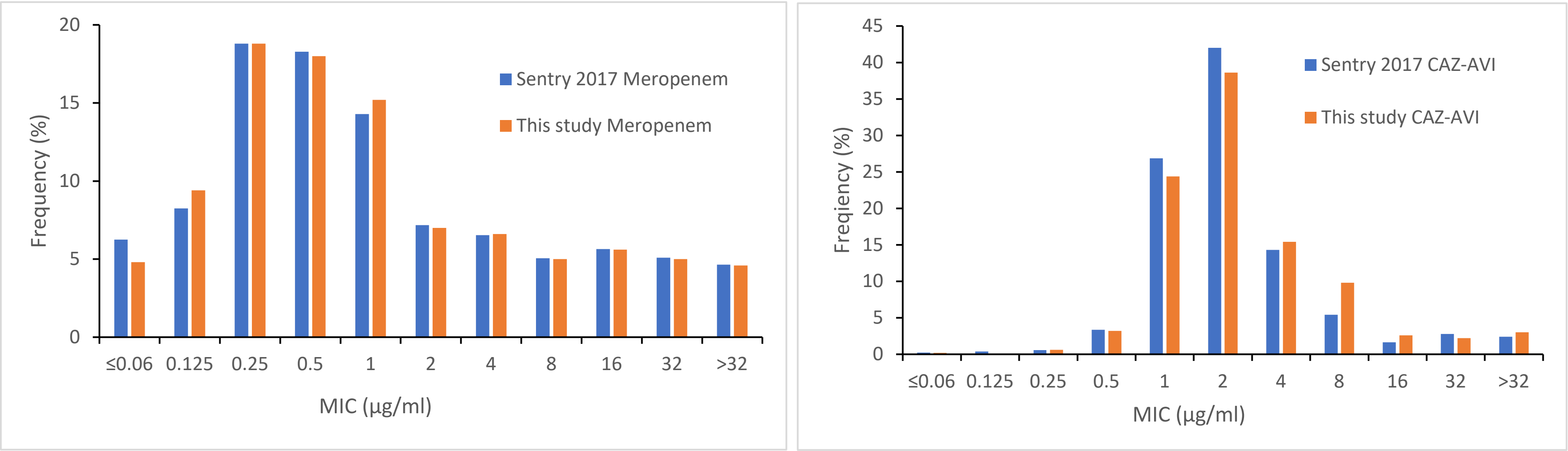

Avibactam was tested at fixed 4 µg/ml

Table S1. Correlation of *mexAB-oprM* expression with aztreonam/avibactam MIC

	MIC (µg/ml)		Relative *mexB* expression*	Amino acid substitution(s) in:		
**Mpex Name**	**Aztreonam alone**	**Aztreonam w/avibactam at 4 µg/ml**		**MexR**	**NalC**	**NalD**
**PAM1020**	4	4	**1.0**	Same as PAO1	Same as PAO1	Same as PAO1
**PA5323**	8	4	**0.7**	V126E	G71E E153Q S209R	Same as PAO1
**PA5317**	4	4	**0.7**	V126E	G71E S209R	Same as PAO1
**PA5297**	4	4	**0.9**	same as PAO1	G71E	Same as PAO1
**PA5300**	4	4	**0.9**	same as PAO1	G71E	Same as PAO1
**PAM3353**	32	2	**0.9**	V126E	G71E, E153Q, S209R	Same as PAO1
**PA5407**	4	2	**1.0**	same as PAO1	G71E S209R	Same as PAO1
**PAM3253**	2	4	**1.4**	same as PAO1	G71E S209R	Same as PAO1
**PAM3218**	8	4	**1.2**	Same as PAO1	G71E	Same as PAO1
**PAM3224**	32	4	**1.3**	same as PAO1	G71E S209R	Same as PAO1
**PAM3235**	8	8	**1.5**	V126E	G71E, A145V, S209R	Same as PAO1
**PAM3377**	8	8	**0.8**	same as PAO1	G71E S209R	Same as PAO1
**PAM3312**	16	8	**1.3**	R83C	G71E S209R	Same as PAO1
**PAM1032**	16	16	**5.4**	**L75R**	Same as PAO1	Same as PAO1
**PAM3244**	32	32	**2.2**	R59G, V126E, V132A	G71E, S209R	Same as PAO1
**PAM3231**	16	16	**4.3**	S26N, D29Y, D34H, E109D, V115L, E145V	K58Q, G71E, S209R	Same as PAO1
**PA5498**	32	32	**4.8**	**R91C** V126E	G71E S209R	Same as PAO1
**PA5456**	64	64	**3.1**	**FS at aa#38**	G71E	Same as PAO1
**PA5397**	64	64	**4.3**	**FS at Q49**	G71E	Same as PAO1
**PA5369**	64	64	**6.8**	**FS at aa#17**	G71E A186T	Same as PAO1
**PA5426**	64	64	**10.5**	**FS at L123**	G71E S209R	Same as PAO1
**PA5459**	64	64	**2.1**	**E109stop**	G71E S209R	Same as PAO1
**PA5352**	65	64	**2.3**	same as PAO1	G71E A145V S209R	Same as PAO1
**PA5338**	>64	>64	**3.0**	**V40stop**	G71E S209R	Same as PAO1
**PA5465**	>64	>64	**6.1**	**L57P V126E**	G71E A145V S209R	Same as PAO1

* Relative to the expression of PAM1020 (PAO1), which is assigned a value of 1. MexR, NalC and NalB are the regulators of the *mexAB-oprM operon* expression. Amino acid substitutions (1-6), frame-shift (FS) mutations and premature terminations that are known to be associated with the increased expression of mexAB-oprM are in bold letters

Table S2. In vitro potency of various beta-lactams combined with QPX7728 at 8 µg/ml against the MBL-negative strains from the challenge panel of *Pseudomonas aeruginosa* according to OprD and Efflux due to MexAB-OprM

	MEM	MEM+ QPX 8 µg/ml	ATM	ATM + QPX 8 µg/ml	PIP	PIP + QPX 8 µg/ml	TOL	TOL+ QPX 8 µg/ml	FEP	FEP+ QPX 8 µg/ml
**No MBL (N=229)**										
**MIC$_{50}$**	16	8	32	16	>64	16	2	1	32	8
**MIC$_{90}$**	64	32	>64	32	>64	64	>64	4	64	16
**% Inhibited***	41.9%	68.1%	17.9%	46.7%	19.7%	69.9%	64.2%	**92.1%**	23.6%	**79.5%**
**OprD Functional (N=47)**										
**MIC$_{50}$**	8	4	32	16	>64	16	4	1	32	8
**MIC$_{90}$**	32	16	>64	32	>64	32	>64	2	64	16
**% Inhibited***	59.6%	**87.2%**	10.6%	38.3%	12.8%	68.1%	61.7%	**97.9%**	12.8%	76.6%
**OprD Non-Functional (N=163)**										
**MIC$_{50}$**	16	8	32	16	>64	16	2	1	32	8
**MIC$_{90}$**	>64	32	>64	64	>64	64	>64	8	64	16
**% Inhibited***	31.9%	58.9%	17.2%	46.0%	17.8%	67.5%	63.8%	**89.6%**	23.9%	**79.1%**
**MexAB-OprM basal level activity (N=77)**										
**MIC$_{50}$**	8	2	16	4	>64	4	2	0.5	16	4
**MIC$_{90}$**	64	4	>64	8	>64	8	>64	1	64	8
**% Inhibited***	70.1%	**96.1%**	44.2%	**96.1%**	35.1%	**97.4%**	67.5%	**96.1%**	39.0%	93.5%
**MexAB-OprM increased activity (N=145)**										
**MIC$_{50}$**	16	8	64	32	>64	16	4	1	32	8
**MIC$_{90}$**	64	32	>64	64	>64	64	>64	8	64	32
**% Inhibited***	25.5%	52.4%	0.0%	17.9%	10.3%	54.5%	60.7%	**89.7%**	15.2%	**72.4%**
**OprD Non-Functional MexAB-OprM increased activity (N=105)**										
**MIC$_{50}$**	32	16	64	32	>64	16	4	1	32	8
**MIC$_{90}$**	>64	32	>64	64	>64	64	>64	16	64	32
**% Inhibited***	13%	40%	0%	18.1%	9.5%	52.4%	58.1%	**86.7%**	13.3%	**72.4%**

MEM, meropenem, QPX, QPX7728, ATM, aztreonam; TOL, ceftolozane; FEP, cefepime; PIP, piperacillin.

*: % inhibited at the following concentrations: meropenem: ≤ 8 µg/ml; meropenem/QPX7728: ≤ 8/8 µg/ml; aztreonam: ≤ 8 µg/ml (FDA susceptible breakpoint); aztreonam/QPX7728: ≤ 8/8 µg/ml; ceftolozane: ≤ 4 µg/ml; ceftolozane/QPX7728: ≤ 4/8 µg/ml; cefepime: ≤ 8 µg/ml (FDA susceptible breakpoint); cefepime/QPX7728: ≤ 8/8 µg/ml.

Table S3. In vitro potency of various beta-lactams combined with QPX7728 at 8 µg/ml against the MBL-positive strains from the challenge panel of *Pseudomonas aeruginosa* according to OprD and Efflux due to MexAB-OprM

	MEM	MEM+ QPX 8 µg/ml	ATM	ATM + QPX 8 µg/ml	PIP	PIP + QPX 8 µg/ml	TOL	TOL+ QPX 8 µg/ml	FEP	FEP+ QPX 8 µg/ml
**MBL (N=61)**										
**MIC_{50}**	>64	32	32	16	>64	16	>64	64	64	32
**MIC_{90}**	>64	>64	>64	64	>64	64	>64	>64	64	64
**% Inhibited***	0.0%	31.1%	23.0%	**42.6%**	3.3%	**72.1%**	3.3%	27.9%	6.6%	36.1%
**OprD Functional (N=11)**										
**MIC_{50}**	32	8	8	8	>64	8	>64	8	64	16
**MIC_{90}**	>64	64	32	32	>64	64	>64	>64	64	64
**% Inhibited***	0.0%	63.6%	54.5%	**72.7%**	0.0%	**81.8%**	9.1%	45.5%	27.3%	45.5%
**OprD Non-Functional (N=48)**										
**MIC_{50}**	>64	32	32	16	>64	16	>64	64	64	32
**MIC_{90}**	>64	>64	>64	64	>64	64	>64	>64	64	64
**% Inhibited***	0.0%	25.0%	16.7%	**37.5%**	4.2%	**70.8%**	2.1%	25.0%	2.1%	35.4%
**MexAB-OprM basal level activity (N=21)**										
**MIC_{50}**	64	4	8	2	>64	4	>64	4	64	8
**MIC_{90}**	>64	64	32	8	>64	16	>64	>64	64	64
**% Inhibited***	0.0%	66.7%	66.7%	**95.2%**	9.5%	**90.5%**	4.8%	52.4%	9.5%	52.4%
**MexAB-OprM increased activity (N=40)**										
**MIC_{50}**	>64	64	32	32	>64	16	>64	>64	64	64
**MIC_{90}**	>64	>64	>64	64	>64	64	>64	>64	64	64
**% Inhibited***	0.0%	12.5%	0.0%	15.0%	0.0%	**62.5%**	2.5%	15.0%	5.0%	27.5%
**OprD Non-Functional MexAB-OprM increased activity (N=33)**										
**MIC_{50}**	>64	>64	32	32	>64	16	>64	64	64	64
**MIC_{90}**	>64	>64	>64	64	>64	64	>64	>64	64	64
**% Inhibited***	0.0%	9.1%	0.0%	12.1%	0.0%	**60.6%**	0.0%	12.1%	0.0%	27.3%

MEM, meropenem, QPX, QPX7728, ATM, aztreonam; TOL, ceftolozane; FEP, cefepime; PIP, piperacillin.

*: % inhibited at the following concentrations: meropenem: ≤ 8 µg/ml; meropenem/QPX7728: ≤ 8/8 µg/ml; aztreonam: ≤ 8 µg/ml (FDA susceptible breakpoint); aztreonam/QPX7728: ≤ 8/8 µg/ml; ceftolozane: ≤ 4 µg/ml; ceftolozane/QPX7728: ≤ 4/8 µg/ml; cefepime: ≤ 8 µg/ml (FDA susceptible breakpoint); cefepime/QPX7728: ≤ 8/8 µg/ml.

MATEIAL AND METHODS

MexB expression studies. Single colonies from overnight grown plates were used to inoculate CaMHB and grown with shaking at 37ºC till OD600=0.6-0.7. Then, 1.5 ml of cultures were collected by centrifugation and total RNA was isolated using Ambion RiboPure-Bacteria RNA Isolation kit (ThermoFisher, Cat# AM1925) according to manufacturer's instructions. Residual DNA in the RNA samples was removed by treatment with DNase I. Reverse transcription (RT) was performed using TaqMan® Reverse Transcriptase Reagents kit (ThermoFisher, cat# N8080234) following the manufacturer's protocol. A mixture of reverse primers for *mexB* and the house-keeping gene *polA*, used as an internal control for quantitative PCR (qPCR) signal normalization, each at 0.5 µM, was used as the RT primers (see Table above). 2µl of RNA samples was added to a total RT reaction volume of 10 µl. qPCR was performed on ABI Prism 7000 Sequence instrument (Applied Biosystems) using SYBR® Select Master Mix (ThermoFisher, cat# 4472919). Each qPCR reaction tube (20 µl total volume) contained 10 µl of SYBR® Select Master Mix (2x), 1 µl of qPCR primer pair mixture resulting in a final concentration of forward and reverse primers at 0.5 µM, and 9 µl of 10-fold dilution (with water) of the RT reaction mixtures. The qPCR was run with the following thermal cycling conditions: 55ºC for 2 min, 95ºC for 5 min, and followed by 40 cycles of 95ºC for 15 sec and 60ºC for 1 min. The qPCR reaction was carried out in duplicate.

The *mexB* gene qPCR results (CT values) were normalized with the housekeeping gene *polA* by subtracting the CT value of *mexB* from the CT value of *polA* of the same RT reaction mixture. To compare the expression of *mexB* between a test strain and the wildtype strain PAM1020 (equivalent to the standard strain PAO1), the normalized CT value of the test strain was subtracted from that of PAM1020, and the difference (ΔCT) was used as a logarithmic power (base=2) to calculate the relative normalized level of mRNA. The average and standard deviation of relative normalized mRNA level in duplicated qPCR reactions from the same RNA sample were calculated.

Primers used in this study

Primer name	Sequence (5' to 3')	Used in
PA-mexR-seq-F	CATGGCCCATATTCAGAACCTG	*mexR* sequencing
PA-mexR-seq-R	CATTCGCCAGTAAGCGGATA	
PA-nalC-seq-F	GAGAAACGCTCTCAGCAAAC	nalC sequencing
PA-nalC-seq-R	TCATCGGGTCCTGAACGAA	
PA-nalD-seq-F	GCAGCATTAGACAAAGGTGG	*nalD* sequencing
PA-nalD-seq-R	CAGGAGGCAATACCATGCAA	
PA-oprD-seq-F	CTATCGCCAAGAAACACTGC	*oprD* sequencing
PA-oprD-seq-R	GCAGAGTAATGAGGAAGACC	
PA-mexB-qF	CTGTTCGCCCAGGTACAGAC	*mexB* RT-qPCR
PA-mexB-qR	CGTCCTTGAAGCTGAAGAAG	
PA-polA-qF	ATCCGAAGAAGCTCAAGGTC	polA RT-qPCR
PA-polA-qR	ATCTGGTCGAAGGTCAGTTG	

REFERENCES

1. Braz VS, Furlan JPR, Fernandes AFT, Stehling EG. 2016. Mutations in NalC induce MexAB-OprM overexpression resulting in high level of aztreonam resistance in environmental isolates of Pseudomonas aeruginosa. FEMS Microbiology Letters 363.
2. Horna G, López M, Guerra H, Saénz Y, Ruiz J. 2018. Interplay between MexAB-OprM and MexEF-OprN in clinical isolates of Pseudomonas aeruginosa. Scientific Reports 8:16463.
3. Quale J, Bratu S, Gupta J, Landman D. 2006. Interplay of efflux system, ampC, and oprD expression in carbapenem resistance of Pseudomonas aeruginosa clinical isolates. Antimicrob Agents Chemother 50:1633-41.
4. Srikumar R, Paul CJ, Poole K. 2000. Influence of Mutations in the mexR Repressor Gene on Expression of the MexA-MexB-OprM Multidrug Efflux System of Pseudomonas aeruginosa. Journal of Bacteriology 182:1410-1414.

5. Saito K, Akama H, Yoshihara E, Nakae T. 2003. Mutations Affecting DNA-Binding Activity of the MexR Repressor of mexR-mexA-mexB-oprM Operon Expression. Journal of Bacteriology 185:6195-6198.

6. Pan YP, Xu YH, Wang ZX, Fang YP, Shen JL. 2016. Overexpression of MexAB-OprM efflux pump in carbapenem-resistant Pseudomonas aeruginosa. Arch Microbiol 198:565-71.
